# Effect of Long-Term Brushing on Deflection, Maximum Load, and Wear of Stainless Steel Wires and Conventional and Spot Bonded Fiber-Reinforced Composites

**DOI:** 10.3390/ijms20236043

**Published:** 2019-11-30

**Authors:** Andrea Scribante, Pekka Vallittu, Lippo V. J. Lassila, Annalisa Viola, Paola Tessera, Paola Gandini, Maria Francesca Sfondrini

**Affiliations:** 1 Unit of Orthodontics and Paediatric Dentistry, Section of Dentistry, Department of Clinical, Surgical, Diagnostic and Paediatric Sciences, University of Pavia, 27100 Pavia, Italy; annalisa.viola01@universitadipavia.it (A.V.); paola.tessera01@universitadipavia.it (P.T.); paola.gandini@unipv.it (P.G.); francesca.sfondrini@unipv.it (M.F.S.); 2 Department of Biomaterial Science and Turku Clinical Biomaterials Centre (TCBC), Institute of Dentistry, University of Turku, 20100 Turku, Finland; pekval@utu.fi (P.V.); liplas@utu.fi (L.V.J.L.); 3 Welfare Division, 20100 Turku, Finland

**Keywords:** fiber, reinforced, composite, FRC, bonding, technique, spot, load, mechanical, deflection, brushing, wear

## Abstract

Fiber-reinforced composite (FRC) retainers are an aesthetic alternative to conventional Stainless Steel splints. They are generally used with a full bonded technique, but some studies demonstrated that they could be managed with a spot bonding technique to significantly decrease their rigidity. In order to propose this FRC spot bonding technique for clinical use, the aim of this study was to evaluate mechanical properties and surface wear of fibers left uncovered. Tests were made by simulating tooth brushing, comparing FRC spot bonding technique splints with stainless steel and FRC traditional technique splints. Specimens were tested both at 0.1 mm of deflection and at maximum load, showing higher values of rigidity for the FRC full bonded technique. After tooth brushing, no significant reduction in values at 0.1 mm deflection was reported, while we found a similar reduction in these values for the Stainless Steel and FRC spot bonding technique at maximum load, and no significant variation for the FRC full bonded technique. SEM images after tooth brushing showed wear for FRC fibers left uncovered, while no relevant wear signs in metal and conventional FRC fibers were noticed. Results showed that FRC spot bonding technique has advantages in mechanical properties when compared to the FRC traditional full bonding technique, also after tooth brushing. However, the surface wear after tooth brushing in the FRC spot bonding technique is considerable and other tests must be performed before promoting this technique for routine clinical use.

## 1. Introduction

Fiber-reinforced composite (FRC) materials were introduced into dentistry about five decades ago, and they were first used as a reconstruction material for fixed dental prostheses (FPDs) [1]. FRC materials have three different components: the matrix or continuous phase, the fibers or dispersed phase, and the interphase [2,3,4]. After light exposure, monomers of the matrix phase convert from fluid to a cross-linked polymer [5]. Fibers are added because of their high strength/weight and stiffness/weight values and good mechanical properties [6,7]. They can be made of different materials (polyethylene, aramid, carbon, glass) [7,8]. Glass fibers are the most common type of fibers that are used in dentistry. These materials have high tensile strength and low extensibility; they are transparent and well suited for applications with high aesthetic demands [9,10]. Clinical applications include: prosthodontics (FDPs and veneers), conservative dentistry (direct restorations), endodontics (root canal anchoring systems), periodontology (periodontal splints), pediatric dentistry (restorations, space maintainers), and orthodontics (fixed retentions) [7,11].

The fixed retention of teeth after orthodontic treatment is often required in order to gain stability and avoid relapse, both in the upper and lower jaw [12,13]. Orthodontic splints are usually made using metallic wires with different sizes, shapes, and diameters [14]. Nowadays, metallic splints are very common, but they cannot be used in patients needing a magnetic resonance scan, as metal can interfere with image quality [15], nor in patients with a nickel allergy or hypersensitivity [16]. Additionally, the increased aesthetic demands in certain cases could lead patients to prefer FRC to metallic fixed retention in order to avoid a decrease in tooth translucency [17].

Some studies have shown that the stiffness of splints with FRC materials is higher than that of splints with metallic ones [18,19,20,21,22]. The high stiffness of FRC materials could reduce physiologic tooth movement and this could increase ankyloses risk, even if this concern has been tested in vivo in a single study that used an animal model [23].

FRC rigidity is probably related to composite bulk that covers fibers totally [24], as suggested by the manufacturer [25,26]. In order to reduce the rigidity of conventional FRC retainers, studies were conducted in order to modify the splinting technique to provide a partial (spot) composite coverage of fibers, only in correspondence with the teeth. This procedure leaves fibers exposed in inter-proximal zones, similarly to metallic splints [7,13]. These reports tested the mechanical properties of the FRC spot bonding technique immediately after bonding, without considering the wear caused by tooth brushing.

Tooth brushing is considered to be a fundamental self-care behavior for the maintenance of oral health and must be performed on fixed retention frameworks.

Abrasion of the composite resins increased linearly with an increasing number of brushing cycles [27]. After some months of tooth brushing, the mechanical properties of FRC materials can change and the surface of fibers with or without composite coverage may wear out.

As no studies have been conducted on this topic, the purpose of the present report was to evaluate the mechanical (load at maximum load) and surface properties (SEM analysis) of different splinting techniques after electrical tooth brushing. The null hypothesis of the present report was that there are no significant differences among the various groups tested in mechanical properties and wear.

## 2. Results

Descriptive statistics are reported in Table 1 and Table 2.

**Figure 1:**
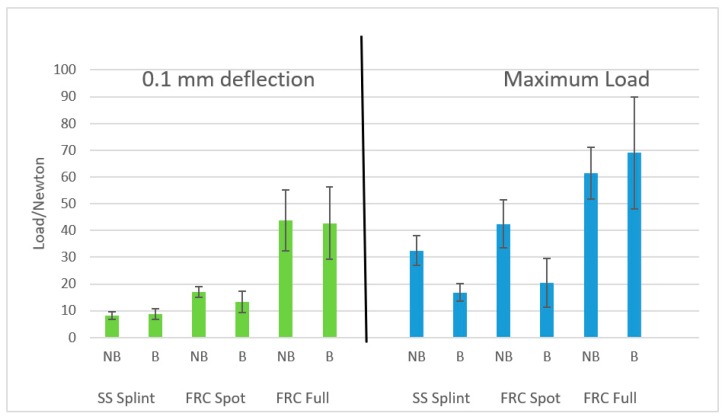
Graphical representation (Mean and SD) of the various groups at 0.1 mm of deflection and at maximum load (NB: not brushed; B: Brushed).

When evaluating values at 0.1 mm of deflection, ANOVA showed significant differences among various groups (*p* < 0.05). The Tukey post hoc test showed that the lowest values (*p* < 0.05) were reported with stainless steel wires (Groups 1 and 2). FRC bonded with a conventional technique (Groups 5 and 6) showed the highest forces (*p* < 0.05). When FRC was tested with the experimental spot bonding technique (Groups 3 and 4), intermediate measures were reported. No significant difference was reported (*p* > 0.05) between not-brushed (Groups 1, 3, and 5) and brushed (Groups 2, 4, and 6) (Figure 1) groups.

Concerning maximum load results, ANOVA showed significant differences among various groups (*p* < 0.05). The post hoc analysis showed that the lowest values (*p* < 0.05) were reported with stainless steel wires (Groups 1 and 2). FRC bonded with a conventional technique (Groups 5 and 6) showed the highest forces (*p* < 0.05). When FRC was tested with the experimental spot bonding technique (Groups 3 and 4), intermediate measures were reported. No significant difference was reported (*p* > 0.05) between not-brushed (Group 5) and brushed (Group 6) FRCs bonded with the conventional technique. On the other hand, both for stainless steel wires (Groups 1 and 2) and for spot bonded FRCs (Groups 3 and 4), the brushed specimens showed significantly lower values (*p* < 0.05) than not brushed ones.

## 3. Discussion

The null hypothesis of the present investigation was rejected. Significant differences were reported among the different tested materials.

There are no studies evaluating the mechanical properties of metallic splints after tooth brushing. In the present report, these retentions were tested and showed no significant difference between not brushed and brushed groups at 0.1 mm of deflection. On the other hand, at maximum load, values significantly decreased after brushing. The lower value of rigidity after brushing may be related to the wear of composite and metal. Previous studies evaluated this variable on composite resins, showing that composite abrasion increased linearly with an increasing number of brushing cycles [27].

Based on the results of the present report, the reduction of rigidity over time seems to not be related to metal abrasion, as SEM microphotographs demonstrated no visible signs of wear. However, the metal splint was covered with composite. The composite surface demonstrated signs of abrasion after SEM evaluation. Therefore, it could be hypothesized that the strength loss could be due to damage of the composite surface.

Some patients cannot wear these devices because of a nickel allergy, as metal release has been demonstrated [28,29]. For these patients, FRCs have been proposed for splinting purposes. Generally, the reinforcement of polymers with continuous fibers is an effective means of developing engineering materials for many applications [1]. Fiber-reinforced plastics are successful primarily because of their high stiffness/weight (specific modulus) and strength/weight (specific strength) when compared with other structural materials [3].

The reinforcement of dental resins with short or long fibers, in contrast to the widely used particulate reinforcement, has been described in the literature for at least 30 years [2]. The filler is made with oriented fibers. The matrix is a light-cured BisGMA (bisphenol A-glycidyl methacrylate). This material is identical to adhesives commonly used in dentistry, thus allowing for high bond strength [30]. The initial stage of polymerization of the matrix makes the framework flexible and adaptable, so that it can be contoured to the teeth before final polymerization [4,12]. The final cure stabilizes the shape and produces optimal mechanical properties [31].

FRCs have the potential to replace metals in clinical prosthodontics and orthodontics [4,8,12,26]. To be considered a viable alternative to existing dental materials, an FRC would first need to be extensively tested both in vitro and in vivo. Previous reports demonstrated the higher stiffness of FRCs compared to conventional metallic splints and wires [18,19,20]. FRC rigidity is mainly related to composite bulk that covers the entire structure once the fiber is placed onto tooth surfaces [7,13].

In the present report, both at 0.1 mm of deflection and at maximum load, significantly higher strength values were recorded for conventional full bonded FRC wires when compared with stainless still wires. The high bend values of the FRC conventional full bonded technique reported in the present investigation is a confirmation of previous studies that showed high rigidity of FRC frameworks [13,18,20,21] and splints [13,19,22] as compared with metallic ones. Ideal retention should allow physiologic tooth movements and the higher rigidity of FRC conventional splints could reduce possible tooth movement. A previous in vivo study (performed on animals) demonstrated that a lack of movement could be related to a higher ankyloses risk [23]. This concern has yet to be fully developed and at present no direct correlation has been verified in human studies.

In order to reduce the high values of rigidity, an FRC spot bonding technique has recently been introduced [7,13] that shows a significant reduction of rigidity both at 0.1 mm of deflection and at maximum load. The present report confirmed these results, as when FRC was tested with the experimental spot bonding technique intermediate measures were reported between metal wires and FRC full bonded splints, with a high reduction of rigidity as compared to the FRC full bonded technique both at 0.1 mm of deflection and at maximum load.

The conventional FRC full bonded technique uses flow composite to cover the entire splint [25]. In the FRC spot bonding technique, the flow composite is placed only in correspondence with the tooth surface, leaving FRC not covered in inter-proximal areas.

Uncovered FRC, like other restorative materials, can be damaged over time. Tooth brushing is a basic oral health practice; however, no other studies to our knowledge evaluated FRC splints after this procedure. Previous authors tested other restorative materials after tooth brushing showing wear effects [32,33].

In the present report, an electrical oscillating rotating toothbrush was used. All toothbrushes have significant plaque removal efficacy, but the magnitude of the reduction was consistently superior for the oscillating rotating power toothbrush compared to either the sonic power or manual brush in all the ’hard-to-clean” region-specific analyses [34,35].

For deflection values, after tooth brushing, when evaluating results at 0.1 mm of deflection, no significant difference was reported between not-brushed and brushed groups for each tested bonding technique. For maximum load values, however, a significant difference was reported between not-brushed and brushed groups of stainless steel spot bonding and FRC spot bonding techniques, while no significant difference was reported between not-brushed and brushed groups of the FRC conventional full bonding technique. The reason for this is the amount of composite is higher and continuous in this technique than in the others. Additionally, evaluating the results that were obtained with fully covered FRC at maximum load, a slight increase of values was reported after tooth brushing. However, the increase was not significant and could be related to the fact that FRC are hand-made frameworks: the amount of composite could be different among various specimens, thus slightly influencing final flexural forces.

**Figure 2:**
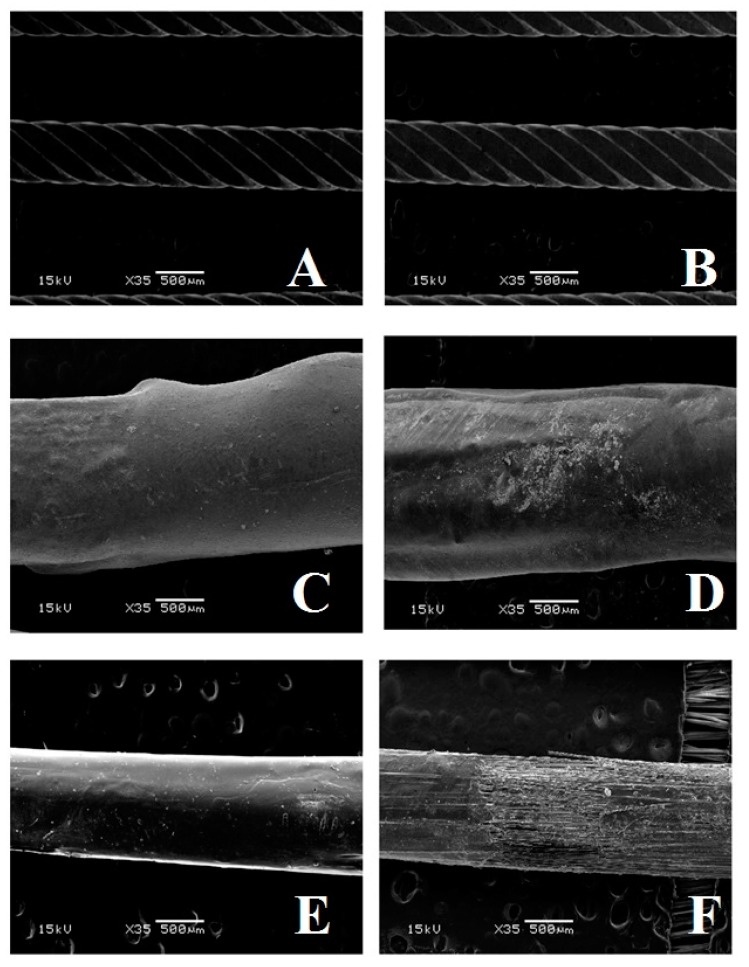
Microphotographs of the various conditions tested at ×35 magnification. (**A**): Flat metallic wire not brushed. (**B**): Flat metallic wire after 26 min of brushing. (**C**): Fiber-reinforced composite (FRC) full bonded and not brushed. (**D**): FRC full bonded after 26 min of brushing. (**E**): FRC spot bonded and not brushed. (**F**): FRC full bonded after 26 min of brushing.

**Figure 3:**
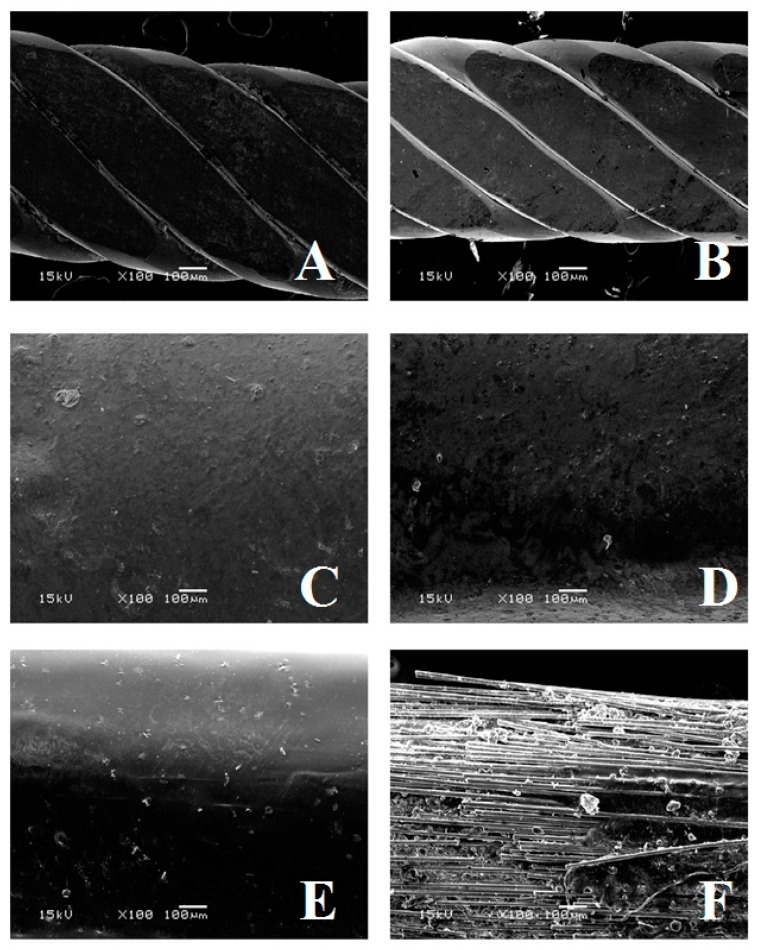
Microphotographs of the various conditions tested at ×100 magnification. (**A**): Flat metallic wire not brushed. (**B**): Flat metallic wire after 26 min of brushing. (**C**) FRC full bonded and not brushed. (**D**): FRC full bonded after 26 min of brushing. (**E**): FRC spot bonded and not brushed. (**F**): FRC spot bonded after 26 min of brushing.

Concerning surface wear, when evaluating scanning electron microphotographs (Figure 2 and Figure 3), the FRC spot bonded technique showed a significantly higher level of visual wear after tooth brushing when compared to the other techniques.

Minimal changes were observed for the metallic retainers. The composite signs of wear that we observed in the FRC full bonded technique are similar to those shown in other studies [32]. At present, no tests have been conducted on FRC surface wear after tooth brushing.

The present study presents some limitations, as only some materials have been taken into consideration, even if in the present market other diameters and shapes are available. Therefore, further in vitro reports are needed in order to evaluate other variables, such as duration, bonding efficiency, and bacterial adhesion of fibers left uncovered, before clinical use.

Moreover, as this was an in vitro study, we could not completely simulate real clinical conditions, so randomized controlled clinical trials would be welcome in order to confirm the results of the present investigation.

## 4. Materials and Methods

In the present in vitro study, flat metallic splints (Straight 8 Lingual Retainer Wire 6’ length, DB Orthodontics, Silsden, United Kingdom) and FRCs (Everstick ORTHO, StickTech, Turku, Finland) were tested. The same operator (AV) performed all of the experimental procedures.

**Figure 4:**
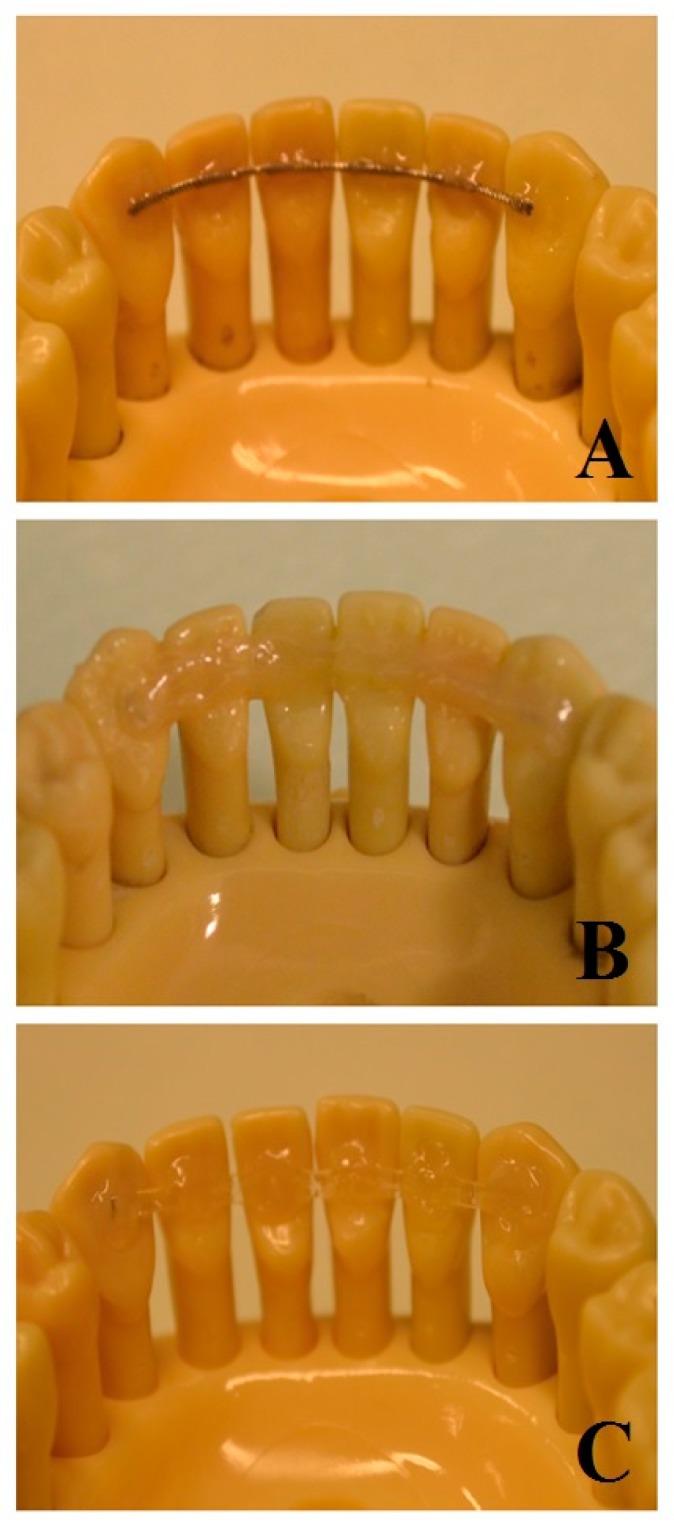
Three different tested materials. (**A**): Flat Metallic wire spot bonded. (**B**): FRC full bonded. (**C**): FRC spot bonded.

Frasaco models (ANA-4, Frasaco GmbH, Tettnang, Germany) were prepared (Figure 4) and splinted with different techniques in order to have six groups of six specimens each:

Group 1: Flat Metallic wire not brushed

Group 2: FRC Full Bonded technique not brushed

Group 3: FRC Spot bonded technique not brushed

Group 4: Flat Metallic wire 26 min brushed

Group 5: FRC Full Bonded technique 26 min brushed

Group 6: FRC Spot Bonded technique 26 min brushed

Frasaco mandible models were splinted by simulating a canine-to-canine splint [36]. Element 3.1 was inserted into the corresponding hole without rigid fixation, thus allowing for vertical movement of the tooth [7]. On the other hand, other acrylic teeth were screwed into their corresponding holes. 

Frasaco models were prepared by cleaning the surface with pumice paste and etched for 20 s (Scotchbond Universal Etchant, 3M, Monrovia, CA, USA). The metallic and FRC splints were bonded to each element from 3.3 to 4.3, with a one-step self-etch 7th generation bonding agent (G-aenial Bond, GC America, Alsip, IL, USA) and subsequently fixed with flow composite (G-aenial Universal Injectable A2, GC America, Alsip, IL, USA).

For the metal and FRC spot bonded groups (Groups 1, 3, 4, and 6), the composite covered the retainer only in correspondence with each tooth, thus leaving the splint exposed in inter-proximal zones. Conversely, composite coverage was performed also in inter-proximal spaces in full-bonded FRC splints (Groups 2 and 5).

All specimens were then light-cured with a halogen lamp (D-Light Pro, GC Europe, Leuven, Belgium) with a light intensity of 1400 mW/cm^2^ and a wavelength range of 430–480 nm for 40 s for each tooth.

All specimens were left for 24 h in water in an incubator at 37 degrees to simulate clinical conditions.

**Figure 5:**
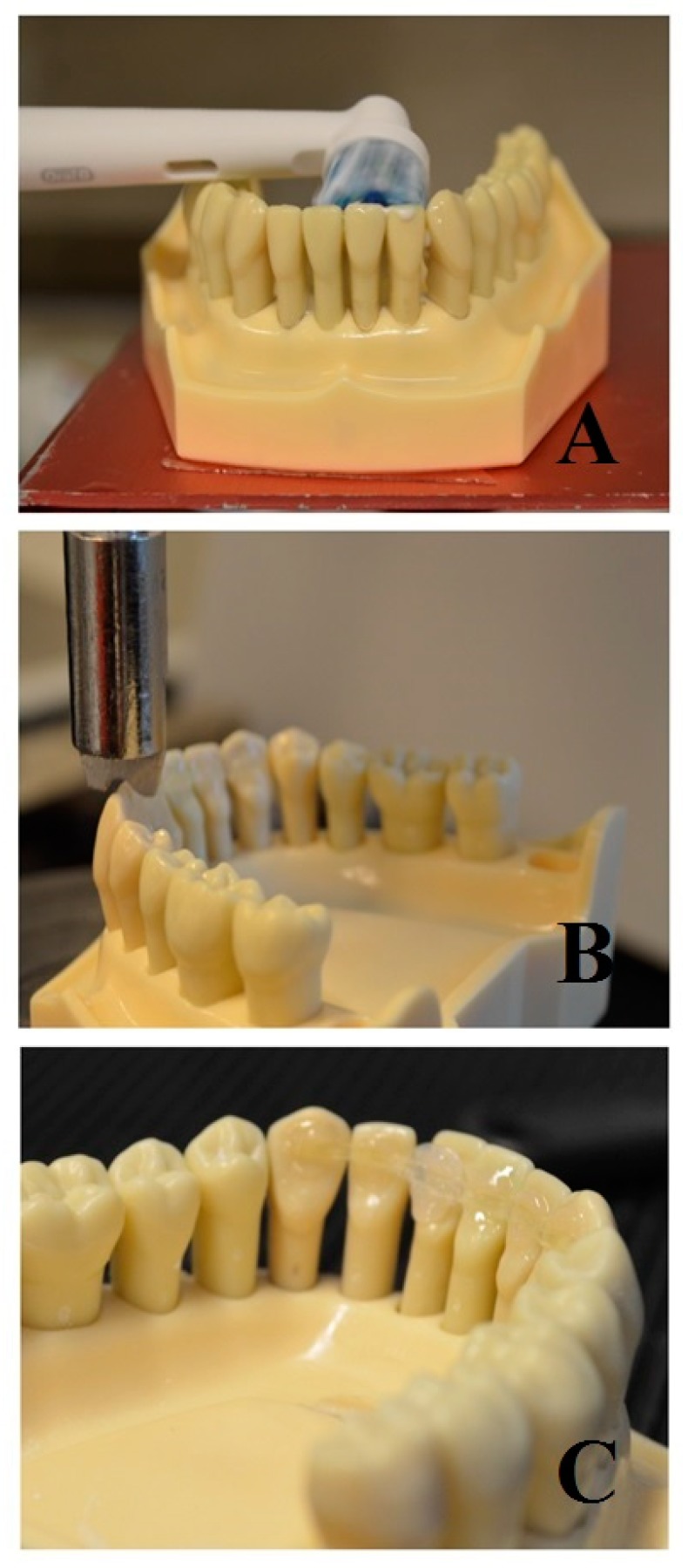
Testing devices. (**A**): Brushing apparatus. (**B**): Mechanical testing machine. (**C**): Specimen after testing.

Subsequently, in the test groups (Groups 4 to 6), the surface of each element 3.1 was subjected to mechanical tooth brushing for 26 min (an Oral B PRO 670 toothbrush with Oral B cross action brush heads, Procter & Gamble, Cincinnati, OH, USA) with a 124 Relative Dentin Abrasion (RDA) toothpaste (MaxWhite white crystals, Colgate-Palmolive, New York, NY, USA) (Figure 5).

Teeth were brushed for 26 min in order to simulate 6 months of tooth brushing. An average brushing time of 2 min was considered two times per day and then divided for the total number of teeth.

(365 days/2) × (2 minutes × 2 times per day/28 teeth)

Subsequently, the strength required to bend the retainer in correspondence of element 3.1 was measured at 0.1 mm of deflection and at maximum load with a universal testing machine (Lloyd LRX; Lloyd Instruments, Fareham, United Kingdom). The crosshead speed was set at 1.0 mm per minute [18,20]. The strength values were recorded in Newton with computer software (Nexygen MT, Lloyd Instruments).

SEM microphotographs (magnification 35× and 100×) were taken using a scanning electron microscope (JEOL 5500, JEOL Ltd., Tokyo, Japan) for all of the tested materials, for both not-brushed and brushed specimens. In order to use the specimens in the scanning electron microscope, they were first treated with a sample sputter coating (BAL-TEC SCD050 Sputter Coater, Capovani Brothers Inc, New York, NY, USA).

Data were submitted for statistical analysis using computer software (R version 3.1.3, R Development Core Team, R Foundation for Statistical Computing, Wien, Austria). Descriptive statistics were calculated for all of the tested groups and reported mean, standard deviation, minimum, median, and maximum values. The Kolmogorov–Smirnov test was used to assess the normality of the data. A multi-factor analysis of variance (ANOVA) and Tukey tests were applied to show differences among groups. Significance for all statistical tests was predetermined at *p* < 0.05.

## 5. Conclusions

The present study demonstrated that the FRC spot bonding technique showed lower values both at 0.1 mm of deflection and at maximum load as compared to the FRC traditional full bonding technique. The experimental framework presented a lack of composite coverage in interproximal areas and, consequently, its design showed important structural differences with the conventional technique. In this case, the oriented fibers that constitute FRC seem to mimic the mechanical behavior of conventional stainless steel splints.

After tooth brushing, the mechanical properties of the FRC spot bonding technique were found to be similar to the properties of Stainless Steel splints.

However, the surface wear of the fibers left uncovered was visually higher than that of the fibers covered with flow composite. Therefore, this concern should be carefully considered when considering clinical applications of this bonding technique.

## Figures and Tables

**Table 1:** Descriptive statistics (N) of the different groups at 0.1 mm of deflection.

Group	Material	Bonding Technique	Brushing	Mean	St Dev	Min	Mdn	Max	Significance *
1	SS	Spot	no	8.26	1.39	6.19	8.90	9.46	A
2	SS	Spot	yes	8.83	1.98	6.57	8.97	11.18	A
3	FRC	Spot	no	16.96	1.89	14.94	16.18	19.33	B
4	FRC	Spot	yes	13.27	4.00	6.27	14.02	17.81	B
5	FRC	Full	no	43.84	11.33	26.14	44.52	58.57	C
6	FRC	Full	yes	42.73	13.41	24.46	43.30	58.29	C

*: means with the same letters are not significantly different.

**Table 2:** Descriptive statistics (N) of the different groups at maximum load.

Group	Material	Bonding Technique	Brushing	Mean	St Dev	Min	Mdn	Max	Significance *
1	SS	Spot	no	32.45	5.59	22.86	35.28	36.87	A
2	SS	Spot	yes	16.83	3.21	12.77	16.09	20.75	B
3	FRC	Spot	no	42.47	8.97	30.37	40.32	53.46	C
4	FRC	Spot	yes	20.50	9.15	7.43	19.07	35.08	B
5	FRC	Full	no	61.36	9.71	46.57	61.39	74.12	C
6	FRC	Full	yes	75.75	21.31	48.95	75.55	108.09	C

*: means with the same letters are not significantly different.
